# ﻿Molecular and morphological evidence support a new species of Rosaceae*Prunus* subg. *Cerasus* from Wuyishan National Park, southeast China

**DOI:** 10.3897/phytokeys.237.115098

**Published:** 2024-01-31

**Authors:** Xiangui Yi, Jingjing Dong, Jie Chen, Huajin Zhou, Tong Wu, Shucheng Gao, Xiangzhen Chen, Meng LI, Xianrong Wang

**Affiliations:** 1 Co-Innovation Center for the Sustainable Forestry in Southern China, College of life sciences, Cerasus Research Center, Nanjing Forestry University, Nanjing 210037, Jiangsu, China Nanjing Forestry University Nanjing China

**Keywords:** molecular phylogeny, morphological comparison Rosaceae, subgenus. *Cerasus*, taxonomy

## Abstract

*Prunustongmuensis*, a new species of cherry blossom, is described and illustrated from Wuyishan National Park, southeast China. This species is characterized by its tubular to nearly bottle-shaped receptacles and dark purple drupes. It can be distinguished from other wild cherry trees by its flowers and leaves, reddish brown young leaves, presence of 1–2 glands at the base of leaves, petioles densely covered with yellowish brown villi, longer pedicels (0.6–2.5 cm), villous pistil, and dark purple drupes. In the present study, we conducted a comprehensive morphological study based on specimens of the new species and its morphologically close species, field observations, and examination of pollen morphology. In addition, our phylogenetic analysis based on the complete plastid genome sequences further confirms the status of the new species and indicates that it is closely related to *Prunusclarofolia*, however, it notably differs in leaf shape, size, petiole villus color, gland location, timing of flower and leaf openings, and reflexed or spread sepals, as well as drupe color.

## ﻿Introduction

PrunusL.subg.Cerasus (Mill.) A. Gray, a member of the family Rosaceae (Li and Bartholomew 2003), is characterized by its densely lenticelled bark, young leaves frequently folded in half, and the presence of glands at the tip of the petiole or along the leaf blade edge (Wang 2014). This subgenus comprises approximately 150 species distributed in the temperate to subtropical zone of the Northern Hemisphere, including Asia, Europe, and North America (Iwatsuki et al. 2001; Li et al. 2022). China has a high species diversity of the subgenus (Zhu et al. 2018), with over 50 species mainly distributed in the western, southwestern, and eastern regions of the country. Most of these species exhibit a broad distribution range and significant morphological variation. Notably, there are distinct differences observed both between different species and between different populations of the same species (Bortiri et al. 2006).

The Wuyi Mountains are situated on the boundary of the provinces Fujian and Jiangxi with an average elevation of 1100 m. The highest point in the region is Huanggang Mountain, reaching an altitude of 2158 m, making the area abundant in resources for cherry blossom (Xie et al. 2007). During 2018–2021, we conducted several botanical expeditions in Wuyishan National Park and its adjacent areas. A population of *Prunus* was discovered in Tongmuguan. However, this species was found to have notable differences from any known species in the genus. In-depth morphological comparison and examination of specimens revealed that the morphology of this species closely resembled that of *Prunusdielsiana* (Schneid.) Yü et Li; however, it notably differs in the location of glands, timing of flower and leaf openings, reflexed or spread sepals, and drupe color (Wang 2014). Further morphological comparisons with other relatives based on herbarium specimens clearly distinguish the Tongmuguan population as a new species due to its distinctive reddish brown young leaves, the presence of 1–2 glands at the base of the leaves, petioles densely covered with yellowish brown villi, longer pedicels (0.6–2.5 cm), pubescent pistil, and dark purple drupes. Hence, we describe, illustrate, and name it as *Prunustongmuensis*, providing a description, accompanied by photographs and a morphological comparison with closely-related species, as well as an exploration of its phylogenetic position within the genus.

## ﻿Materials and methods

### ﻿Morphological study

Morphological observations were conducted based on living plants in the field and dried specimens in herbaria. A total of 20 specimens were collected from seven species, of which two were the new species and 18 specimens from six closely related species. The closely related species are *Prunusclarofolia* (Schneid.) Yü et Li, *P.dielsiana* (Schneid.) Yü et Li, *P.discoidea* Yü & Li, *P.pseudocerasus* (Lindl.) G. Don, *P.conradinae* (Koehne) Yü et Li and P.×subhirtella (Miq.) Sok. The material for morphological study is listed in Table 1. Measurements were conducted manually with rulers or using ImageJ software (Version 1.54b, Bethesda, MD, USA, Rasband 1997–2017). Morphological comparison was carried out among six closely related species based on 18 specimens deposited in herbaria or digital specimens provided by the National Plant Specimen Resource Center, NPSRC (available at https://www.cvh.ac.cn/ accessed 8 March 2019), Global Biodiversity Information Facility (available at https://www.gbif.org/ accessed 9 April 2019), and JSTOR (available at https://plants.jstor.org/ accessed 20 May 2019). Two specimens of the new species were deposited in the Herbarium of Nanjing Forestry University (Voucher specimens *X.G. Yi-201832301*; *X.G. Yi-201832302* (NF)). An identification key of PrunusL.subg.Cerasus (Mill.) for seven species is also provided.

**Table 1.** Species names and voucher specimen information.

Species name	Voucher specimen	Locality
*Prunusclarofolia*	Chen Ze-Ying PE01802945(PE)	Si Chuan, China
	Zhao Qing-sheng & Tan Zhong-ming CDBI0045472(CDBI)	Si Chuan, China
	Xiao Shun-chang CDBI0045468(CDBI)	Si Chuan, China
*Prunusdielsiana*	He Xian-yu NAS00357009(NAS)	An Hui, China
	C.T.Hwa NAS00357021(NAS)	Si Chuan, China
	H.Migo NAS00357093(NAS)	Zhe Jiang, China
*Prunusdiscoidea*	H.Migo NAS00357008(NAS)	Jiang Xi, China
	Li Pan CSH0073332(CS)	Zhe Jiang, China
	Zhang Fang-gang ZMNH0061126(ZMNH)	Zhe Jiang, China
*Prunuspseudocerasus*	J. I. Jeon et al. PE01928048(PE)	Si Chuan, China
	Lin Qin-Zhong CSFI011626(CSFI)	Hu Nan, China
	Tan Ce-ming SZG00026422	Jiang Xi, China
*Prunusconradinae*	Zhou Shi-liang PE2062176(PE)	Yun Nan, China
	T.T.Yu et H.T.Hsai PE01296356(PE)	Gui Zhou, China
	Zhang Dai-gui JIU23231 (JIU)	Hu Bei, China
*Prunus×subhirtella*	Chen Zhi-Yuan CCAU0009236(CCAU)	Hu Bei, China
	C.T.Hwa NAS00358355(NAS)	Si Chuan, China
	Zhang Dai-gui JIU25570(JIU)	Hu Nan, China
*Prunustongmuensis*	Xiangui Yi(NF)	Fu Jian, China
	Xiangui Yi(NF)	Fu Jian, China

The observation of pollen morphology analysis was carried out using pollen samples of specimen *X.G. Yi-201832302* collected from Wuyishan National Park. Mature and well-developed pollen grains were selected for observation. The morphology of pollen grains was scanned and photographed using an electron microscope (ZEISS EVO LS10, Germany) after being sputter-coated with gold.

### ﻿Phylogenetic study

**Figure 1.**
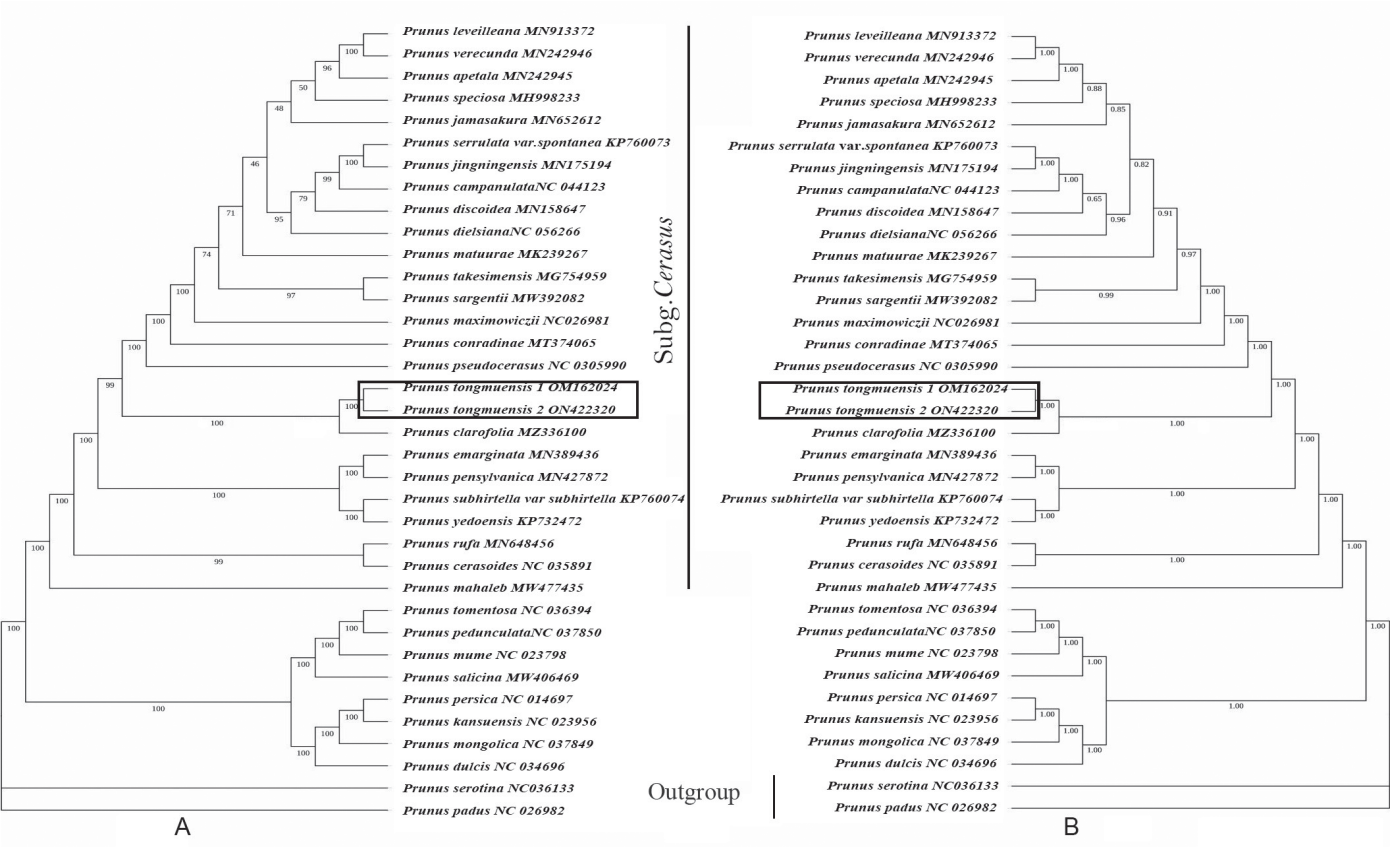
Maximum Likelihood (ML) tree (**A**) and Bayesian inference (BI) tree (**B**) of cherry blossom inferred from the plastid genome. The numbers associated with branches are maximum likelihood bootstrap (MLBS) values of **A** and Bayesian posterior probabilities (PP) of **B**.

Genomic DNA was extracted from fresh leaves of the new species from the specimens *X.G. Yi-201832301* and *X.G. Yi-201832302* (NF) using the DNA extraction kit DP305 (Tiangen Biotechnology (Beijing) Co., LTD.) following the manufacturer’s instructions. DNA quality was measured using a NanoDrop 2000 spectrophotometer (NanoDrop Technologies; Thermo Fisher Scientific, Inc., Wilmington, DE, USA). The qualified DNAs (≥50 ng) were sent to Novogene Bioinformatics Technology Co., Ltd. (Beijing, China) for paired-end (PE) library construction and genome-skimming sequencing. The generated reads were assembled using the GetOrganelle pipeline (Jin et al. 2020). The genome annotation was performed with CpGAVAS (Liu et al. 2012), then the inverted repeat (IR) boundaries were manually adjusted and confirmed using Geneious prime version 2021.0.4 (https://www.geneious.com/). In total, two plastid genomes of the new species were assembled and annotated. In order to determine the phylogenetic position of this species in PrunusL.subg.Cerasus (Mill.), complete plastid genomes of 34 plastid genomes were downloaded from NCBI and aligned with the two plastid genomes of the new species to reconstruct the phylogenetic trees with *P.serotina* and *P.padus* as outgroups (Fig. 1). The 36 plastid genomes were initially aligned using MAFFT version 7 (Katoh and Standley 2013), and then manually checked and edited using PhyloSuite version 1.2.2. Maximum likelihood (ML) analyses were conducted using IQ-tree version 1.6.12 (Trifinopoulos et al. 2016) with 10,000 ultrafast bootstrap (UFBS) replicates. The Bayesian Information Criterion (BIC) was employed to calculate the best fitting substitution models using PhyloSuite version 1.2.2. (Lu et al. 2018; Fu et al. 2020; Medeiros et al. 2020). Bayesian inference (BI) analysis was carried out using MrBayes version 3.2.2 (Ronquist et al. 2012). The Markov chain Monte Carlo analysis was executed for 2,000,000,000 generations, with one cold and three heated chains, each starting with a random tree, and sampled at every 1000 generations. Convergence of runs was accepted when the average standard deviation (*d*) of split frequencies was < 0.01. The first 25% of the trees were discarded as burn-in, and the remaining trees were used to construct majority-rule consensus trees. The final trees obtained from ML and BI analyses were visualized using FigTree v.1.4.2 (Rambaut 2009).

## ﻿Results and discussion

### ﻿Morphological study

The morphological study revealed distinctive characteristics of the new species *P.tongmuensis*, including elongated oval leaves with doubly serrated edges lacking glands. Additionally, the leaves are densely covered with fine villi on both surfaces. The young leaves display a reddish-brown color and are adorned with small yellow villi on the petioles. There are one to two glands situated at the base of each leaf blade. The bracts of the new species appear oblong or fan-shaped, distinguished by their short pedicels. Its hypanthium displays a tubular-campanulate form, as its sepals vary between triangular or long lanceolate shapes with entire margins. Notably, the style slightly surpasses the stamens in length. Based on these traits, it is inferred that the new species pertains to PrunusSubg.Cerasus. Morphologically, the new species bears the closest resemblance to *P.dielsiana* in terms of leaf shape. However, the new species can be easily distinguished from *P.dielsiana* by having 1–2 glands at the base of the leaves and the sepals are not reflexed (Table 2). In addition, we compared the new species to other morphologically similar species and revealed significant differences among them (see the key).

**Table 2.** Diagnostic macro-morphological characteristic of *P.tongmuensis*, *P.clarofolia* and *P.dielsiana*.

	*P.tongmuensis*	*P.clarofolia*	*P.dielsiana*
Leaf shape	no glands at the end of teeth	tooth end with small glands or glands not obvious	obvious glands at the end of tooth
Leaf size(cm)	4–10 /2–4	3–6 /2–4	6–14 /2.5–4.5
Petiole villi color	Yellow	white	white
Glands	1–2 glands at leaf base	1–2 glands on the petiole	1–3 glands on the petiole
Inflorescence	Flowers 1-(2, 3)-4, flowers opening at same time as leaves	Flowers 2–4(-5), flowers opening at same time as leaves	Flowers 3–6, flowers opening before leaves
Hypanthium	tubular-campanulate	campanulate	campanulate
Sepal	erect or spread	reflexed	reflexed
drupe color	ripening dark purple	ripening red	ripening red

### ﻿Key to the new species and its morphologically similar species in the genus

**Table:** 

1	Sepals reflexed	**2**
–	Sepals erect or spread	**4**
2	Petals rounded and obtuse, hypanthium glabrous, style base sparsely pubescent, bracts with conical or capitate glands at tooth ends, stipules linear	**1. *P.clarofolia***
–	Petals sharply lobed or conspicuously concave, hypanthium outside pilose, style glabrous; stipules narrowly banded	**3**
3	Flowers opening before leaves	**2. *P.discoidea***
–	Flowers opening at same time as leaves	**3. *P.dielsiana***
4	Leaf margins bluntly notched and double serrated, hypanthium tubular, drupe ripening red	**5**
–	Leaf edges sharply double serrated, hypanthium suburceolate, drupe ripening black or dark purple	**6**
5	Hypanthium outside sparsely pilose, flowers white	**4. *P.pseudocerasus***
–	Hypanthium smooth and glabrous, flowers white, pink or red	**5. *P.conradinae***
6	Flowers opening before leaves, petiole with 1–3 glands, densely white pubescent	**6. *P.subhirtella***
–	Flowers opening at same time as leaves, leaf blade base with 1–2 glands, petiole densely yellow pilose	**7. *P.tongmuensis***

### ﻿Plastid genome structure of *Prunustongmuensis*

**Figure 2.**
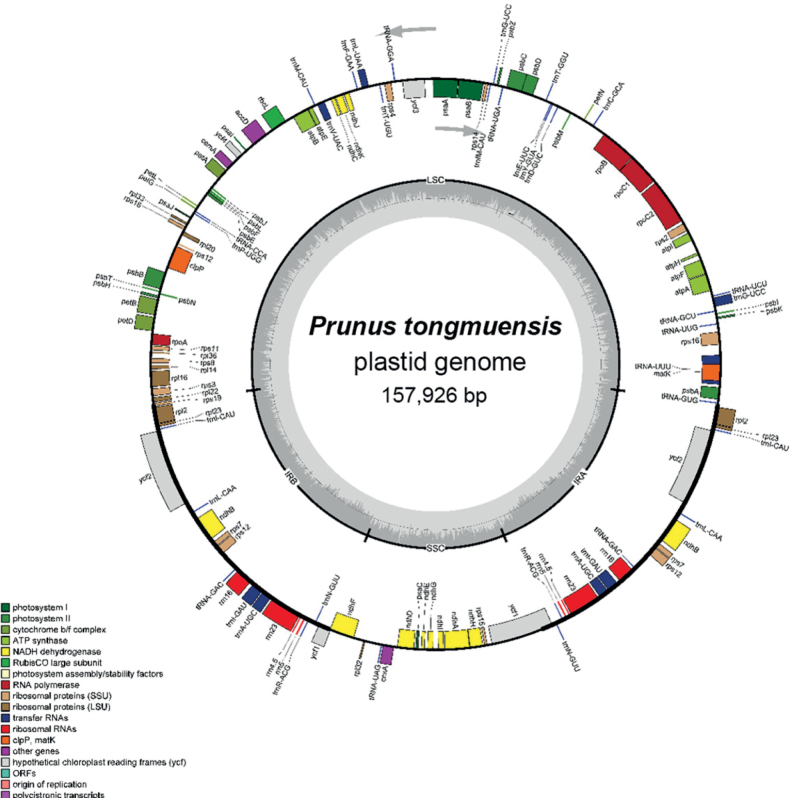
Plastid genome map of *Prunustongmuensis*.

The plastid genome of *P.tongmuensis* (voucher specimen *X.G. Yi-201832301*) exhibits a ring tetrad structure typical for higher plants. The total length of the genome is 157,926 bp, consisting of a large single-copy region (LSC) with the length of 86,025 bp, a small single-copy region (SSC) with the length of 19,117 bp, and two inverted repeat regions with combined length of 26,392 bp. The total GC content is 36.7%, while the AT content is 63.3% (Fig. 2). The plastid genome of *P.tongmuensis* totally contains 130 genes, which can be categorized into three groups: 85 protein-coding genes (PCGs), 37 transfer RNA (tRNA) genes, and 8 ribosomal RNA (rRNA) genes. 21 genes are duplicated in the two inverted repeat regions.

Among the 130 genes, 81 genes can be attributed to two categories: genes involved in photosynthesis, such as Photosystem I, Photosystem II, Cytochrome b/f 6 complex, ATP synthase; and NADH dehydrogenase and genetically related genes, including RubisCO large subunit, RNA polymerase, Ribosomal proteins-SSU, Ribosomal proteins-LSU, transfer RNAs, and Ribosomal RNAs. Additionally, there are 11 genes, which consist of *clpP*, *matk*, and genes related to the hypothetical chloroplast reading frames.

### ﻿Molecular phylogenetic study

The alignment of the plastid genomes was 164,917 bp long. The model TVM+F+R2 for ML analysis and GTR+F+I+G4 for BI analysis was used according to the Bayesian information criteria (BIC). Our results show that Prunussubg.Cerasus is resolved as a clade with strong support values (BS = 99%, PP = 1), which is consistent with previous studies (Shi et al. 2013; Shen et al. 2023). Two accessions of the new species *P.tongmuensis* were well resolved as a distinct clade, which is sister to the species *P.clarofolia* (BS = 100%, PP = 1) (Fig. 1).

### ﻿Taxonomic treatment

#### ﻿ Prunus tongmuensis

Taxon classificationPlantaeRosalesRosaceae

X.G.Yi & X.R.Wang sp. nov.

FDC2BB86-26A1-5CBD-A316-3C867364A16B

urn:lsid:ipni.org:names:77335470-1

Figs 3 , 4

##### Diagnosis.

This species closely resembles *P.dielsiana* in leaf shape, however, it can be distinguished by no glands at the end of teeth (obvious glands at the end of tooth in *P.dielsiana*), petiole yellow villous (petiole white villous in *P.dielsiana*), 1–2 glands at leaf base (1–3 glands on the petiole in *P.dielsiana*), Flowers 1–4 (2, 3), flowers opening at same time as leaves (flowers 3–6, flowers opening before leaves in *P.dielsiana*), sepal erect or spread (sepal reflexed in *P.dielsiana*), ripening dark purple (ripening red in *P.dielsiana*).

**Figure 3.**
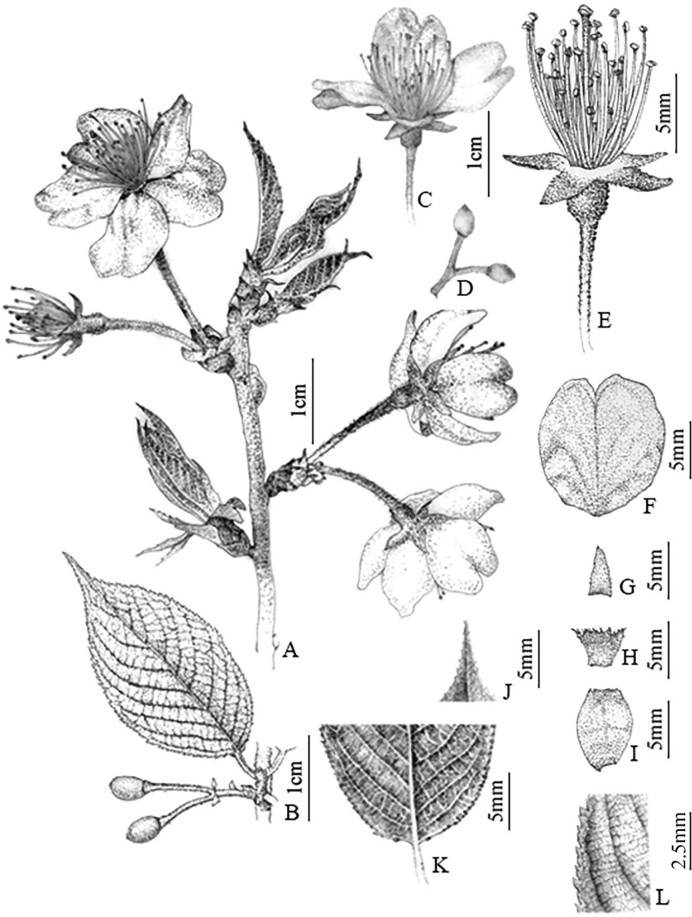
*Prunustongmuensis* X.G.Yi & X.R.Wang **A** flowering branch **B** fruiting branch **C** flower **D** fruit **E** pistil and stamen **F** petal **G** sepal **H** bract **I** involucral bract **J** leaf apex **K** two glands at the base of leaf **L** teeth.

##### Type.

China. Fujian Province: Tongmuguan, Wuyishan National Park, 27°74.91'N, 117°67.49'E, elev. ca. 728 m, 10 March, 2018, *X.G. Yi 201832301*NF-201832302 (Holotype).

**Figure 4.**
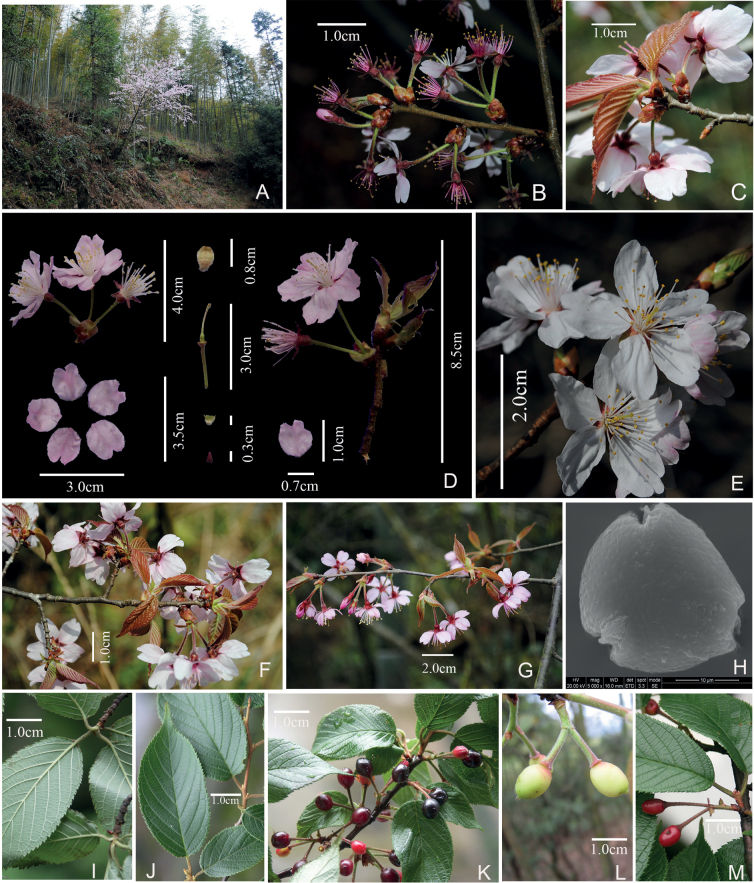
*Prunustongmuensis* X.G.Yi et X. R.Wang **A** habitat **B–G** flowering branch **H** pollen grain **I, J** leaves **K** fruiting branch **L** young fruit branch **M** dark purple fruits.

##### Description.

Trees or shrubs, 3–10 m tall. Bark grayish brown. Young branchlets yellow-green, densely yellow villous. Winter buds ovoid, 2–4 mm. Stipules narrowly lorate, slightly shorter than petiole, caducous, margin glandular-serrate. Petiole 5 × 8 mm, densely covered with yellow villi. Leaf blade obovate, elliptic, or obovate-oblong, 4–10 × 2–4 cm, base rounded to broadly cuneate, margin biserrate or sharply serrulate, teeth with no gland; main and secondary veins densely covered with villous, secondary veins 7–12. Inflorescences umbellate, 1–4-flowered, involucral bracts brown, long elliptic, 6–8 mm long, ca. 3 mm in width, adaxially densely covered with appressed villous; bracts ovate, 1–3 mm in diam., margin strongly fimbriate, fimbria with a long-stalked gland. Flowers opening before leaves or nearly at the same time. Pedicel 0.5–1(–2) cm, spreading white villous. Hypanthium tubular-campanulate, 3–5 × 3–4 mm, outside densely villous, pubescent. Sepals usually reddish, triangular, 0.4–5.5 cm, erect, margin entire, apex acute to obtuse. Petals white or pink, ovate to obovate, apically entire or emarginate. Stamens 35–40, shorter than petals. Style as long as stamens, glabrous, stigma disciform. Drupe dark purple, ovoid, 1–1.2 × 0.5–0.8 cm; endocarp ovoid, 0.6–0.8 × 0.4 cm, deeply furrowed and pitted on the lateral sides, apex obtuse. Flowers Mar.–Apr., fruits in May.

##### Etymology.

Referring to the locality (Tongmuguan) where this new species was found.

##### Distribution and habitat.

This species is currently known only from Wuyishan National Park, Fujian and Jiangxi Province. This species grows in various habitats such as the margins of evergreen broad-leaved forests, valleys, or roadsides, at an altitude of 600–1000 m.

## ﻿Conclusions

We have described and illustrated a new species of PrunusL.subg.Cerasus (Mill.) within the family Rosaceae in Tongmuguan, Tongmu Village situated on the border of Jiangxi and Fujian provinces in China. Additionally, we have presented evidence for its phylogenetic position through the whole plastid genome data. Following comprehensive field research, we have determined that *Prunustongmuensis* is confined to a narrow range within Tongmuguan, located in the break pass of the Wuyi Mountains. On the edge of the forest, four large populations of nearly 60 individuals, each with about 15 individuals, were observed scattered. It is quite interesting that morphologically, this species bears the closest resemblance to *P.dielsiana*, with significant differences in the location of glands, timing of flower and leaf openings, reflexed or spread sepals, and drupe color. Phylogenetically, the new species is closely linked to *P.clarofolia*; however, it notably differs in leaf shape, size, petiole villus color, gland location, timing of flower and leaf openings, and reflexed or spread sepals, as well as drupe color. Our study not only contributes to the diversity of Prunussubg.Cerasus species in China but also underscores the importance of conducting a comprehensive survey of biodiversity in the Jiangxi and Fujian provinces and the Wuyi Mountains.

## Supplementary Material

XML Treatment for Prunus tongmuensis

## ﻿Additional information

### Author ORCIDs

Xiangui Yi https://orcid.org/0000-0003-3123-9076

Jingjing Dong https://orcid.org/0000-0001-5269-4778

Meng Li https://orcid.org/0000-0003-2085-3388

Xianrong Wang https://orcid.org/0000-0002-7940-909X

### Data availability

All of the data that support the findings of this study are available in the main text.

## References

[B1] Bortiri E Heuvel BV Potter D (2006) Phylogenetic analysis of morphology in *Prunus* reveals extensive homoplasy. Plant Systematics and Evolution 259(1): 53–71. 10.1007/s00606-006-0427-8

[B2] Fu LF Liao R Lan DQ Lan DQ Wen F Liu H (2020) A new species of *Chrysosplenium* (Saxifragaceae) from Shaanxi, north-western China. PhytoKeys 159: 127–135. 10.3897/phytokeys.159.56109 32973392 PMC7486313

[B3] Iwatsuki K Boufford DE Ohba H (2001) Flora of Japan. Science Press 2: 435–148.

[B4] Jin JJ Yu WB Yang JB Song Y de Pamphilis CW Yi TS Li DZ (2020) GetOrganelle: A fast and versatile toolkit for accurate de novo assembly of organelle genomes. Genome Biology 21(1): e241. 10.1186/s13059-020-02154-5 PMC7488116 32912315

[B5] Katoh K Standley DM (2013) MAFFT multiple sequence alignment software version 7: Improvements in performance and usability. Molecular Biology and Evolution 30(4): 772–780. 10.1093/molbev/mst010 23329690 PMC3603318

[B6] Li CL Bartholomew B (2003) *Cerasus* in Flora of China. Science Press 9: 404–420.

[B7] Li M Song YF Sylvester SP Sylvester SP Wang XR (2022) Comparative analysis of the complete plastid genomes in PrunussubgenusCerasus (Rosaceae): Molecular structures and phylogenetic relationships. PLOS ONE 17(4): e0266535. 10.1371/journal.pone.0266535 PMC8985974 35385520

[B8] Liu C Shi LC Zhu YJ Chen HM Zhang JH Lin XH Guan XJ (2012) CpGAVAS, an integrated web server for the annotation, visualization, analysis, and GenBank submission of completely sequenced chloroplast genome sequences. BMC Genomics 13(1): e715. 10.1186/1471-2164-13-715 PMC3543216 23256920

[B9] Lu Z Li Y Yang X Liu J (2018) *Carpinustibetana* (Betulaceae), a new species from southeast Tibet, China. PhytoKeys 98: 1–13. 10.3897/phytokeys.98.23639 PMC5943419 29750069

[B10] Medeiros H de Carvalho Lopes J Acevedo-Rodríguez P Forzza RC (2020) A new species of *Thinouia* (Paullinieae, Sapindaceae) from the Amazon and its phylogenetic placement. PhytoKeys 165: 115–126. 10.3897/phytokeys.165.57341 33192150 PMC7642129

[B11] Rambaut A (2009) FigTree 1.3.1. http://tree.bio.ed.ac.uk/software/figtree

[B12] Ronquist F Teslenko M van der Mark P Ayres DL Darling A Höhna S Larget B Liu L Suchard MA Huelsenbeck JP (2012) MrBayes 3.2: Efficient Bayesian phylogenetic inference and model choice across a large model space. Systematic Biology 61(3): 539–542. 10.1093/sysbio/sys029 22357727 PMC3329765

[B13] Shen X Zong W Li Y Liu X Zhuge F Zhou Q Zhou S Jiang D (2023) Evolution of Cherries (PrunusSubgenusCerasus) based on chloroplast genomes. International Journal of Molecular Sciences 24(21): we15612. h 10.3390/ijms242115612 PMC10650623 37958595

[B14] Shi S Li J Sun J Yu J Zhou SL (2013) Phylogeny and classification of *Prunussensulato* (Rosaceae). Journal of Integrative Plant Biology 55(11): 1069–1079. 10.1111/jipb.12095 23945216

[B15] Trifinopoulos J Nguyen L von Haeseler A Minh Q (2016) W-IQ-TREE: A fast online phylogenetic tool for maximum likelihood analysis. Nucleic Acids Research 44(W1): W232–W235. 10.1093/nar/gkw256 PMC4987875 27084950

[B16] Wang XR (2014) An illustrated monograph of cherry cultivars in China. Science Press 12: 24–28.

[B17] Xie CP Yi XG Wang XR (2007) Population structure and dynamics of Cerasussubhirtellavar.ascendens in Wuyi Mountain, Fujian. Shihezi Daxue Xuebao. Ziran Kexue Ban 25(6): 732–736.

[B18] Zhu H Yi XG Zhu SX Wang HC Wang XR (2018) Application of the molecular marker technology to *Cerasus* Mill. (Rosaceae). World Forestry Research 31: 16–24.

